# Measuring Adolescent Boys' Physical Activity: Bout Length and the Influence of Accelerometer Epoch Length

**DOI:** 10.1371/journal.pone.0092040

**Published:** 2014-03-18

**Authors:** Taren Sanders, Dylan P. Cliff, Chris Lonsdale

**Affiliations:** 1 University of Western Sydney, School of Science and Health, Penrith, Australia; 2 University of Wollongong, Interdisciplinary Educational Research Institute, School of Education, Wollongong, Australia; The Scripps Research Institute, United States of America

## Abstract

**Objectives:**

Accurate, objective measurement is important for understanding adolescents' physical activity (PA) behaviour. When using accelerometry to objectively measure PA, a decision must be made regarding how frequently data is recorded (i.e., epoch length). The purpose of this study was to examine i) PA bout length, and ii) the effect of variations in accelerometer epoch length on PA estimates during physical education (PE) and leisure time in adolescent boys.

**Design:**

Cross-sectional study.

**Methods:**

Year 9 boys (N = 133; mean age ±SD  = 14.36±0.48 years) wore accelerometers during two PE lessons, and for a period of seven consecutive days. Data were reintegrated from 1s into longer periods of 2, 5, 10, 30, and 60 seconds. ANOVAs were used to test for differences in PA estimates between epochs in leisure time and PE.

**Results:**

The mean length of vigorous PA (VPA) bouts was 3.5±2.0 seconds for PE and 2.5±1.7 seconds for leisure time, and mean length of moderate PA (MPA) bouts was 2.3±0.5 seconds for PE and 2.9±0.5 seconds for leisure time. During PE, estimates of MVPA, MPA, and light PA (LPA) increased as epoch increased from 1 second to 60 seconds, while VPA and sedentary behaviour estimates decreased. During leisure time, estimates of all PA intensities decreased as epoch increased from 1 second to 60 seconds, with the exception of sedentary behaviour, which increased as epoch length increased.

**Conclusion:**

The context in which PA occurs can influence PA bout length measurement and the effect of variations in epoch length on PA estimates. Researchers measuring PA with accelerometry should be conscious of the possible influence of context on PA estimates.

## Introduction

Regular physical activity (PA) during adolescence provides a number of health benefits [1]–[3]. However, PA declines rapidly during this time [4], [5], making adolescence a priority period for PA promotion and research. Accurately quantifying PA is necessary to conduct meaningful research involving adolescents. Due to their ability to provide objective, time-stamped data on the intensity, frequency, and duration of PA with low participant burden [6], [7], accelerometers have become the instrument of choice for assessing habitual PA among adolescents.

In order to accurately measure PA using accelerometry, a variety of decisions must be made, including the frequency at which data is recorded, known as an epoch [8], [9]. An epoch represents the length of time that activity ‘counts’- a measure of activity magnitude - are summed before being stored by the accelerometer. The choice of epoch should be largely determined by the PA bout length (i.e. time spent in a single PA intensity [10], [11]) of the population being studied [8]. When PA bout length is shorter than the epoch length, there is potential for misclassification, causing the estimations of PA to become skewed. Therefore, an understanding of PA bout length is important when assessing accelerometer epoch effects.

To the authors' knowledge, only two studies have measured PA bout length among young people. Both were conducted in a leisure time context with children aged 6–10 years [10], [11]. Bailey et al. [10] used direct observation during leisure time to show that 6–10 year-old children's PA was highly intermittent, with a median bout length for all PA intensities of ≤6 s. Baquet et al.[11] used accelerometers with 2 s epochs to measure bout length in children aged 8–10 years and reported similar results to Bailey et al. More than 95% of vigorous or very vigorous PA lasted <10 s, with average bout length for all intensities ≤9 s. These two studies are routinely cited as a justification for using a short epoch [12], [13]. However, there is a need for evidence from other populations, including adolescents, whose PA behaviour might potentially be less sporadic and intermittent than that of children.

Varying the epoch length has been shown to influence estimations of youth PA volume and intensity [9], [14], [15]. In an adolescent sample, Edwardson and Gorely [8] found that using a 5, 15, or 30 s epoch produced significantly different estimates of moderate-to-vigorous PA (MVPA) compared to a 60 s epoch. When using the same equipment, comparing similar epoch lengths, and similar samples, Nilsson et al. [14] demonstrated that estimates of 7 to 8 year-old children's MVPA decreased as epoch length increased from 5 s to 60 s during physical education (PE), while McClain et al. [9] found that estimates of 10 to 11 year-old children's MVPA increased as epoch length increased during leisure time. McClain et al. hypothesised that the difference in results might have occurred because structured PA, such as PE, may have shorter, more intense bouts of PA compared to unstructured PA occurring during leisure time. To date, this hypothesis has not been tested using data gathered from the same sample.

The first objective of this study was to compare adolescent boys' PA bout length in two PA contexts; leisure time and physical education lessons. The second objective was to compare the effect of varying accelerometer epoch length on estimates of MVPA, vigorous PA (VPA), moderate PA (MPA), light PA (LPA), and sedentary behaviour in both contexts.

It was hypothesized that: 1) the mean bout lengths of all PA intensities would be longer in a leisure time context, compared to the PE context; 2) estimates of MVPA, VPA, and MPA would be higher using a short epoch length compared to a longer epoch length, in both contexts; and 3) due to shorter, more intense PA bouts occurring in PE lessons, the impact of varying the epoch length on PA intensity estimation would be more pronounced in PE than in leisure time.

## Methods

A sample of 133 male Year 9 students (mean age ±SD  = 14.36±0.48 years; mean BMI±SD  = 22.36±3.83) were recruited from six PE classes within a boys Catholic school in Sydney, Australia. To be eligible to participate, students were required to have no existing conditions that would prevent their participation in PA.

Before PA data were collected, trained research assistants used a portable stadiometer to measure height to the nearest 0.5 cm (Surgical and Medical Products No. 26SM, Medtone Education Supplies, Melbourne, Australia), and a set of digital scales to measure weight to the nearest 0.1 kg (UC-321, A&D Company LTD, Tokyo, Japan). Body mass index (BMI; kg/m^2^) was calculated for each student.

Prior to data collection, students were taught the correct procedure for fitting the accelerometer. They were instructed to ensure that the device faced upwards, was worn securely against the top of the right hip, and was worn during all waking hours, except for periods when the device may get wet (e.g., showering or swimming) or increase injury risk due to involvement in contact sports. Then they participated in a familiarisation PE lesson with the device attached. The purpose of this lesson was to reduce any reactivity effects that might result from wearing the accelerometer. Data from the familiarisation lesson was not used in any analysis.

Students' PA during PE was then measured over the next two PE lessons, both of which focused on soccer. The students' regular teacher conducted all lessons as per usual practice (i.e., no special instructions were provided by the research team). Between these two data collection lessons, students' leisure time PA was measured over a period of seven consecutive days. To be included in the analyses, students were required to wear the accelerometer for at least ten hours on at least four days, including one weekend day [7], [16], and participate in at least one of the PE lessons. To increase compliance, students who met this requirement were given a $20 gift card [17].

Actigraph GT3X accelerometers (Actigraph; Pensacola, FL) were used to assess PA. Each accelerometer was attached to the students' hip at the top of the right iliac crest by means of a cotton elastic belt, as per best practise protocols and the manufacturer's recommendations [9], [16], [18]. Before all PA assessments, the accelerometers were initialised to record tri-axially using a 1s epoch.

At the end of each PE lesson, and at the conclusion of the seven-day period, data from the accelerometers were downloaded to a computer. A purpose-designed spreadsheet was used to process the accelerometer data. This process involved: (a) identifying and removing periods of non-wear time, defined as 20 min or more of consecutive zeros [19]; (b) reintegrating the original 1 s epoch into longer periods of 2, 5, 10, 30, and 60 s epochs by summing 1 s epochs into longer lengths (e.g., 1 s + 1 s  =  2 s epochs); (c) classifying PA into MVPA, VPA, MPA, LPA, and sedentary behaviour; and (d) identifying and quantifying the number and length of each PA bout. PA intensities were classified using the Freedson et al. [20], [21] metabolic equivalents (METs) prediction equation, with ≥4 METs the threshold for MPA, and ≥7 METs the threshold for VPA [22]. Sedentary behaviour was defined as ≤100, and LPA was defined as greater than sedentary and less than MPA. These thresholds exhibit good classification accuracy among adolescents [22] and have been used extensively [23]. As per the Freedson et al. equation requirements, only vertical axis data were used. As the Freedson et al. equation is based on 60 s epochs, division was used to convert cut points for epochs shorter than 60 s (e.g., 60 s cut points/30 = 2 s cut points). This method has been adopted in multiple studies [8], [9], [24]

Data from the spreadsheet was exported into SPSS software package (version 20) for analyses. Independent samples t-tests were used to check for differences in BMI, height, and weight between those students with and without valid accelerometer data. When testing for PA bout length differences between contexts, a MANOVA analysis was not possible due to the high correlations between MVPA and VPA, MVPA and MPA, and between MVPA and sedentary behaviour, violating the assumption of linearity[25]. As a result, a series of repeated measure t-tests was conducted, with bout length at each PA intensity as the dependent variable in each test.

To investigate epoch effects, a series of two-way repeated-measure ANOVAs was used to test differences in total time spent in each PA intensity for each epoch (1, 2, 5, 10, 30, and 60 s). To begin, one ANOVA was conducted for each dependent variable (time spent in MVPA, VPA, MPA, LPA, and sedentary). The independent variables in each ANOVA were context (2 levels) and epoch length (6 levels). Following significant results, separate ANOVAs were run for each context separately, with Bonferroni *post hoc* t-tests used to identify differences in PA estimates when ANOVA identified significant differences. The significance level for all tests was set at *p*<.05. Cohen's [26] guidelines were used to interpret standardised mean difference effect sizes (*d):* small >0.2, medium >0.5, and large >0.8.

### Ethics Statement

The University of Western Sydney Human Research Ethics Committee granted approval for this study. Prior to data collection, students' parents, physical education teacher, and school principal provided voluntary informed written consent, and students provided voluntary written assent. Data from this study is available upon request.

## Results

Of the 180 students enrolled in the six PE classes, 133 (74%) volunteered to participate. The total number of participants with valid datasets included in the analysis was 74 (56%; mean age ±SD: 14.3±0.46; mean BMI±SD: 22.38±3.86). Students who did not have a complete dataset were not significantly different from students who did have complete data for weight (*p = *.80), height (*p = *.66), or BMI (*p* = .92). Participants with a valid dataset averaged 5.23±1.21 valid days, with an average wear time of 13.47±1.94 hours/day. Compared with leisure time data, proportions of time spent in PA were higher in the PE context. Participants averaged 36.0±12.3% lesson time in MVPA, 15.2±5.8% VPA, 20.7±8.2% MPA, 33.1±8.8% LPA, 30.9±14.3% sedentary time during PE lessons. In comparison, participants with a valid data set averaged 6.3±2.0% wear time in MVPA, 1.4±1.0% VPA, 4.9±1.4% MPA, 11.4±2.8% LPA, and 82.3±4.1% sedentary behaviour during leisure time.

### Bout Length

**Figure 1:**
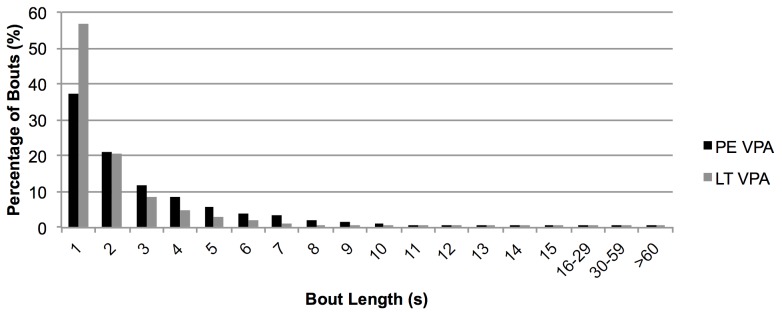
Percentage of vigorous physical activity (VPA) bouts by duration during physical education (PE) and leisure time (LT).

When using a 1 s epoch length, the mean PA bout lengths were ≤5.6 s for all PA intensities in both contexts, excluding sedentary behaviour (Table 1). MVPA, VPA, and LPA bout lengths were significantly longer in the PE context, compared to the leisure time context. This difference was large for MVPA (*d* = 1.06) and LPA (*d* = 0.96), and medium for VPA (*d* = 0.51). In contrast, MPA (*d* = 1.16) and sedentary (*d* = 3.24) bout lengths were longer in the leisure time context than PE. In both the PE and leisure time contexts, >95% of VPA and MPA bouts were less than 9 s in duration. Further, >58% of VPA and MPA were accumulated in 1–2 s bouts and frequency of bouts decreased as bout length increased (Table 2). This decrease is shown in more detail for VPA in Figure. 1.

**Table 1:** Comparison of Physical Activity Bout Lengths by Context.

					95% Confidence Interval			
	Physical Education Lesson Mean Seconds (SD)	Leisure Time Mean Seconds (SD)	Mean Difference Between Contexts (PE – LT) Seconds (SD)	*p* (2-Tailed)	Lower	Upper	*t (df)*	Cohen's *d*
MVPA	5.6 (1.7)	4.1 (0.9)	1.5 (1.9)	<.001	1.0	1.9	6.7 (73)	1.06
VPA	3.5 (2.0)	2.5 (1.7)	1.0 (2.7)	.003	0.3	1.6	3.1(73)	0.51
MPA	2.3 (0.5)	2.9 (0.5)	−0.6 (0.7)	<.001	−0.8	−0.5	−7.6 (73)	1.16
LPA	2.9 (0.7)	2.5 (0.2)	0.5 (0.6)	<0.01	0.3	0.6	−6.9 (73)	0.96
SED	5.6 (3.1)	24.5 (7.6)	−18.9 (8.3)	<0.01	−20.8	−16.9	−19.5 (73)	3.24

*Note.* MVPA  =  moderate to vigorous physical activity; VPA  =  vigorous physical activity; MPA  =  moderate physical activity; LPA  =  light physical activity; SED  =  sedentary time; PE  =  physical education; LT  =  leisure time; SD  =  standard deviation, *d*  =  standardised difference.

**Table 2:** Proportion of Physical Activity Bouts by Length and Intensity.

	Average Percentage (%) of Bouts (SD)									
	MVPA		VPA		MPA		LPA		SED	
Bout length (*s*)	PE	LT	PE	LT	PE	LT	PE	LT	PE	LT
1	31.9 (7.4)	37.3 (9.8)	54.6 (8.3)	46 (3.6)	56.7 (7.2)	55.2 (3.9)	42.6 (6.4)	48.5 (1.9)	39.8 (8.7)	24.8 (2.4)
2	16.2 (4.9)	20.9 (5.5)	20.6 (4.3)	19.5 (1.4)	20.5 (2.4)	19.5 (1.1)	20.9 (3.0)	22.6 (0.9)	16.3 (3.9)	12.2 (1.2)
3	10.3 (3.2)	12.0 (4.3)	9.8 (2.8)	9.9 (1.0)	8.6 (1.8)	8.4 (0.9)	11.8 (2.5)	11.1 (0.6)	10.0 (3.2)	8.0 (0.7)
4	6.9 (2.8)	8.4 (3.7)	5.2 (2.2)	5.7 (0.6)	4.7 (1.4)	4.6 (0.7)	7.2 (2.1)	6.0 (0.5)	6.4 (2.5)	5.8 (0.4)
5	4.9 (2.1)	5.9 (3.4)	3.0 (1.8)	3.7 (0.5)	2.8 (1.2)	2.8 (0.5)	4.8 (1.7)	3.6 (0.4)	4.6 (2.3)	4.5 (0.4)
6	4.2 (2.0)	3.9 (2.6)	1.8 (1.2)	2.7 (0.5)	1.9 (0.9)	1.9 (0.5)	3.5 (1.4)	2.3 (0.4)	3.7 (2.1)	3.7 (0.3)
7	3.6 (2.0)	3.2 (2.6)	1.1 (1.1)	2.0 (0.4)	1.3 (0.7)	1.3 (0.3)	2.2 (1.3)	1.5 (0.3)	2.7 (1.8)	3.1 (0.3)
8	3.1 (1.8)	2.0 (1.6)	0.8 (0.7)	1.6 (0.4)	0.8 (0.5)	1.0 (0.3)	1.8 (1.1)	1.1 (0.2)	2.1 (1.7)	2.6 (0.2)
9	2.4 (1.7)	1.5 (1.5)	0.6 (0.7)	1.2 (0.3)	0.6 (0.5)	0.8 (0.2)	1.1 (0.9)	0.8 (0.2)	1.8 (1.4)	2.2 (0.2)
10	2.2 (1.3)	1.2 (1.4)	0.6 (0.7)	1.0 (0.3)	0.4 (0.3)	0.6 (0.2)	0.8 (0.8)	0.6 (0.2)	1.4 (1.3)	2.0 (0.2)
11	1.7 (1.2)	0.8 (1.0)	0.3 (0.6)	0.8 (0.2)	0.3 (0.3)	0.5 (0.2)	0.6 (0.6)	0.4 (0.1)	1.1 (1.1)	1.5 (0.2)
12	1.6 (1.1)	0.7 (0.9)	0.3 (0.5)	0.7 (0.2)	0.2 (0.2)	0.4 (0.2)	0.6 (0.6)	0.3 (0.1)	1.0 (1.0)	1.6 (0.2)
13	1.4 (1.1)	0.5 (0.8)	0.3 (0.4)	0.5 (0.2)	0.2 (0.2)	0.3 (0.1)	0.4 (0.4)	0.2 (0.1)	1.0 (1.1)	1.4 (0.2)
14	1.0 (0.9)	0.4 (0.7)	0.2 (0.3)	0.4 (0.2)	0.1 (0.1)	0.3 (0.1)	0.2 (0.4)	0.2 (0.1)	0.7 (0.9)	1.3 (0.2)
15	1.1 (0.9)	0.1 (0.4)	0.2 (0.3)	0.4 (0.2)	0.1 (0.1)	0.2 (0.1)	0.3 (0.3)	0.1 (0.1)	0.7 (0.9)	1.1 (0.1)
16–29	5.4 (3.2)	0.9 (1.6)	0.5 (0.8)	2.5 (0.9)	0.5 (0.4)	1.5 (0.6)	1.2 (1.5)	0.6 (0.3)	4.2 (2.3)	9.3 (1.0)
30–59	1.8 (2.4)	0.1 (0.4)	0.1 (0.4)	1.0 (0.5)	0.1 (0.2)	0.6 (0.3)	0.1 (0.2)	0.1 (0.1)	1.8 (1.8)	6.7 (1.3)
60+	0.3 (0.7)	0.4 (2.4)	0 (0)	0.5 (0.3)	0.1 (0.2)	0.2 (0.2)	0.0 (0.1)	0 (0)	0.8 (1.3)	8.1 (2.6)

*Note.* MVPA  =  moderate to vigorous physical activity; VPA  =  vigorous physical activity, MPA  =  moderate physical activity; LPA  =  light physical activity; SED  =  sedentary behaviour.

### Epoch Effects

The ANOVA results indicated a significant difference between PE and leisure time for time in MVPA (Wilks' λ = .11; F (5, 69)  = 109.84; *p*<0.001), VPA (Wilks' λ = .26; F (5, 69)  = 39.19; *p*<0.001), MPA (Wilks' λ = .24; F (5, 69)  = 44.45; *p*<0.001), LPA (Wilks' λ = .03; F (5, 69)  = 347.65; *p*<0.001) and sedentary behaviour (Wilks' λ = .04; F (5, 69)  = 333.53; *p*<0.001). In the PE context, different estimates of PA at all intensities, and sedentary behaviour, were observed for all epoch lengths, with the exception of 1 s vs. 2 s epochs for VPA (*p* = 1.00) and 30 s vs. 60 s epochs for LPA (*p* = 0.61; Table 3). Estimated time spent in MVPA, MPA, and LPA increased as epoch length increased. The opposite result was observed for VPA and sedentary behaviour, with longer epochs generating smaller estimates compared with shorter epochs.

**Table 3:** Differences in Physical Activity Estimates by Epoch in the Physical Education Context.

Physical Activity Intensity	Epoch (s)	Mean minutes per lesson (SD) (*j*)	Mean Difference Between Epochs (min)					
			1 (*i*)	2 (*i*)	5 (*i*)	10 (*i*)	30 (*i*)	60 (*i*)
MVPA	1	11.6 (3.9)	_					
	2	11.7 (4.0)	0.2**	_				
	5	12.2 (4.1)	0.6**	0.4**	_			
	10	12.9 (4.3)	1.3**	1.1**	0.7**	_		
	30	13.7 (4.8)	2.1**	1.9**	1.5**	0.8**	_	
	60	14.3 (5.7)	2.8**	2.6**	2.2**	1.5**	0.7*	_
VPA	1	4.9 (1.8)	_					
	2	4.9 (2.0)	0.00	_				
	5	4.7 (2.1)	−0.2**	−0.2**	_			
	10	3.9 (2.3)	−1.0**	−1.0**	−0.8**	_		
	30	2.3 (2.3)	−2.6**	−2.6**	−2.4**	−1.6**	_	
	60	1.2 (1.9)	−3.7**	−3.7**	−3.5**	−2.7**	−1.1**	_
MPA	1	6.7 (2.6)	_					
	2	6.9 (2.6)	0.2**	_				
	5	7.5 (2.7)	0.8**	0.7**	_			
	10	9.0 (3.1)	2.3**	2.1**	1.5**	_		
	30	11.4 (3.9)	4.7**	4.5**	3.9**	2.4**	_	
	60	13.1 (5.1)	6.5**	6.3**	5.6**	4.2**	1.8**	_
LPA	1	10.7 (2.8)	_					
	2	11.7 (3.0)	1.1**	_				
	5	13.0 (3.3)	2.4**	1.3**	_			
	10	13.9 (3.5)	3.2**	2.2**	0.9**	_		
	30	15.3 (4.4)	4.6**	3.6**	2.3**	1.4**	_	
	60	15.8 (5.5)	5.2**	4.1**	2.8**	1.9**	0.5	_
SED	1	10.2 (5.1)	_					
	2	8.9 (4.9)	−1.3**	_				
	5	7.2 (4.5)	−3.0**	−1.7**	_			
	10	5.6 (4.0)	−4.6**	−3.3**	−1.6**	_		
	30	3.1 (3.2)	−7.0**	−5.8**	−4.0**	−2.4**	_	
	60	1.9 (2.8)	8.3**	−7.0**	−5.3**	−3.7**	−1.2**	_

*Note.* MVPA  =  moderate to vigorous physical activity; VPA  =  vigorous physical activity, MPA  =  moderate physical activity; LPA  =  light physical activity; SED  =  sedentary time. Differences calculated as *i*-*j*. *p<.05, **p<.01.

A different trend was noted in the leisure time context (Table 4). Different estimates of MVPA, VPA, LPA, and sedentary behaviour were observed for all epoch lengths, with the exception of 1 s vs. 2 s epochs for MVPA (*p* = .20). Fewer differences were observed for MPA, as there were no significant differences between estimates from epochs of 1 s, 2 s, 5 s, and 10 s. However, 1, 2, 5, and 10 s epochs all produced significantly different estimates of MPA compared to 30 and 60 seconds. Epoch lengths of 30 and 60 s also produced significantly different estimates of MPA. For MVPA, VPA, and MPA, longer epochs provided smaller estimates compared to shorter epochs, while the opposite was true for LPA and sedentary behaviour.

**Table 4:** Differences in Physical Activity Estimates by Epoch in the Leisure Time Context.

PA Intensity	Epoch (sec)	Mean minutes per day (SD) (*j*)	Mean Difference Between Epochs (min)					
			1 (*i*)	2 (*i*)	5 (*i*)	10 (*i*)	30 (*i*)	60 (*i*)
MVPA	1	51.9 (18.3)	_					
	2	49.0 (15.5)	−2.9	_				
	5	47.2 (15.6)	−4.7**	−1.8**	_			
	10	45.4 (15.8)	−6.5**	−3.6**	−1.8**	_		
	30	41.5 (16.2)	−10.3**	−7.5	−5.7	−3.9	_	
	60	37.8 (16.1)	−14.1**	−11.2**	−9.4**	−7.6**	−3.8**	_
VPA	1	11.6 (11.2)	_					
	2	10.0 (7.6)	−1.6**	_				
	5	8.5 (7.3)	−3.0**	−1.5**	_			
	10	7.1 (7.0)	−4.5**	−2.9**	−1.5**	_		
	30	4.6 (6.4)	−7.0**	−5.4**	−3.9**	−2.4**	_	
	60	3.4 (6.2)	−8.2**	−6.6**	−5.1**	−3.7**	−1.2**	_
MPA	1	40.3 (11.9)	_					
	2	39.00 (11.6)	−1.3	_				
	5	38.6 (11.8)	−1.7	−0.4	_			
	10	38.3 (12.3)	−2.0	−0.7	−0.3	_		
	30	36.9 (13.7)	−3.4*	−2.1*	−1.7*	−1.4**	_	
	60	34.4 (14.3)	−5.9**	−4.6**	−4.3**	−4.0**	−2.5**	_
LPA	1	92.9 (22.7)	_					
	2	109.9 (28.3)	17.0**	_				
	5	140.4 (35.0)	47.5**	30.5**	_			
	10	169.0 (41.0)	76.1**	59.1**	28.5**	_		
	30	218.4 (50.6)	125.5**	108.5**	78.0**	49.5**	_	
	60	254.0 (57.7)	161.1**	144.1**	113.6**	85.0**	35.6**	_
SED	1	676.0 (90.5)	_					
	2	658.2 (93.2)	−17.8**	_				
	5	629.4 (96.5)	−46.6**	−28.8**	_			
	10	602.6 (99.6)	−73.4**	−55.6**	−26.8**	_		
	30	556.9 (105.1)	−119.1**	−101.3**	−72.5**	−45.7**	_	
	60	525.0 (109.2)	−151.0**	−133.2**	104.4**	−77.6**	−31.9**	_

*Note.* MVPA  =  moderate to vigorous physical activity; VPA  =  vigorous physical activity, MPA  =  moderate physical activity; LPA  =  light physical activity; SED  =  sedentary time. Differences calculated as *i-j*. *p<.05, **p<.01.

## Discussion

This study compared PA bout lengths, and the effect of accelerometer epoch length on estimates of PA, in PE and leisure time contexts among adolescent boys. It was hypothesised that PA bout length would be shorter in the PE context compared to the leisure time context. Contrary to this hypothesis and previous findings [9], PA bout lengths were significantly shorter in the leisure time context than the PE context. Additionally, PA bout length in this study differed to that observed in previous bout length research in children. The mean leisure time PA bout length in this study was 2.9 s and 2.5 s for MPA and VPA, respectively. These bout lengths are considerably shorter than the 9 s and 4.7 s for MPA and VPA, respectively, observed in previous research with 8–12 year-old children during leisure time [11]. Given that PA bout length is generally assumed to be shorter for children than adults [27], [28], it is surprising that PA bout length in the current sample of adolescents was shorter than that of the younger children in previous research. These unexpected results may be due to slight differences in methodology between this study and previous bout length research. The lowest possible length of an observed PA bout for each study is the length of the observation period (i.e., 2 s for Baquet et al. [11], 3 s for Bailey et al. [10], and 1 s for the present study), which may explain the difference in results. In addition, Baquet et al. [11] defined MPA as ≥3 METs, whereas in this study cut-points corresponding to ≥4 METs were used, as these have been shown to have excellent classification accuracy [22]. The lower threshold for MPA may also explain the difference in results. Future research should examine bout length using multiple measures, including a criterion measure (e.g., high frequency direct observation), to determine the most accurate estimate of PA bout length.

The frequency of bouts decreased as bout length increased, indicating that the majority of adolescent males' PA bouts are short (1–2 s) in duration. The frequency of bouts and the trend of frequency decreasing by bout length in this study are similar to those of Baquet et al. [11] among 8–10 year old children and Obeid et al. [29] among 3–5 year old preschoolers. This finding further suggests that short epoch lengths (i.e., 2 s or less) may be most appropriate for accurately measuring adolescent PA.

It was hypothesised that estimates of MVPA, VPA, and MPA would decrease as epoch length increased. The results of this study supported this hypothesis for leisure time PA; however, estimated MVPA and MPA increased with epoch length in the PE context, while VPA decreased. Decreases in estimates of MVPA, VPA, and MPA as epoch length increased in the leisure time context were likely due to short bouts of PA being misclassified as LPA, as indicated by LPA increasing as epoch length increased. This finding is consistent with the results of several previous studies conducted with child samples during leisure time [24], [30], [31]. There were fewer significant differences between estimates of MPA for epochs in leisure time (14.6%) than in any other context or intensity, consistent with previous literature [8], [14], [29], where little or no effect on MPA was observed. This finding strengthens the notion that it is the more intense PA that is most likely to be affected when different epoch lengths are used.

Increases in estimates of MVPA, MPA and LPA in the PE context are likely the result of VPA being misclassified as lower intensity PA. Other studies have also found that estimates of MPA and MVPA increased as epoch length increased, including Rowland et al. 's [15] study conducted during school hours, and McClain et al. 's [9] study conducted during a PE lesson. The differences in epoch effects between contexts identified in this study indicates that the context in which PA is measured can moderate the estimate of PA, especially for VPA.

Estimates of VPA were impacted by epoch length more than estimates of other PA intensities in both contexts. When compared to estimates produced by the 60 s epoch, estimates of VPA produced by the 1 s epoch were more than four times larger in the leisure time context, and more than three times larger in the PE context. This finding supports previous literature, which suggests that VPA is largely underestimated when using longer epoch lengths [29]–[31].

To the authors' knowledge, this is the first study to examine 1 s epochs in an adolescent sample. While the physiological benefits of brief bouts of VPA may be limited, the total amount of VPA may be important in reducing obesity and adiposity levels, due to the increase in energy expenditure compared to MPA[32]. Further, there is some evidence which suggests brief bouts of VPA may decrease cardiometabolic risk factors [33], [34], and improve bone health [35]. Studies investigating PA behaviour among preschoolers have used 3 s epochs in order to avoid underestimating MVPA[29]. It is possible that the same logic may need to be applied to adolescent samples. Therefore, the accurate measurement of short bouts of PA, in particular VPA, is important.

It was hypothesised that shorter PA bout lengths would cause more pronounced differences between estimates of PA. However, a greater number of significant epoch effects were noted in the PE context compared to the leisure context, despite mean bout length being shorter in the leisure time context. However, it should be noted that while the *number* of epoch effects was larger in the PE context, the actual *size* of the epoch-related differences in PA estimates were also greater in the PE context compared to the leisure time context. Indeed, the difference in estimates between 1 s and 60 s epochs for VPA and MPA in the PE context was proportionally larger than the equivalent estimates in leisure time. This finding suggests that PA bout length may not be the only factor that influences epoch effects. Both the duration and the frequency of PA bouts may have a role in influencing estimates of PA. Frequent short bouts of PA may still be captured by an accelerometer when using a longer epoch length, if they occur close together.

The differences in estimates of PA between epoch lengths indicate that comparisons between studies using different epoch lengths are limited. Specifically, comparisons of studies using differing epoch lengths should be avoided for all epoch lengths and intensities, with the exception of 1 and 2 s epochs for VPA. In the leisure time context, comparisons between studies using different epoch lengths should be avoided entirely for VPA, and studies using 1, 2, 5, or 10 s epochs should not be compared to studies using 30 or 60 s epochs when examining MPA. Further, comparisons should not be made between studies using 1, 2, or 5 s epochs and 10 or 60 s epochs, 1 s and 30 s epochs, 2 s and 5 s epochs, or 10 s or 60 s epochs for MVPA in a leisure time context. These findings are similar to, and build upon, the findings of Edwardson and Gorely [8]. This study aimed to build on the research of Edwardson and Gorely in two ways: 1) by examining epoch lengths <5 s, and 2) by trying to explain the reason why shorter epochs generate different results to longer ones (i.e., by examining bout length). Edwardson and Gorely did not conduct analysis of bout length, which is a novel aspect of this study. While our study methodology does not allow us to draw conclusions as to which epoch length is most accurate, we do aim to provide evidence as to why short bout lengths may produce more accurate measures of PA (i.e., due to short bout lengths).

Limitations of this study should be noted. The sample consisted only of male students from a single school. It is possible that results for bout length may differ for females of the same age, given that it is known PA bout length is slightly longer in female children than males[11]. However, it is not known if this difference in bout length occurs in adolescents. Additionally, this study examined bout length during PE for only one sport (i.e., soccer). It is not known how bout length would differ during other activities. Further, with growing support for shorter epoch lengths [16], [36], specific cut points and MET equations for different epoch lengths need to be more thoroughly understood. Current practise involves using cut points or MET equations designed for 60 s epochs, but adjusted to suit shorter epoch lengths. The accuracy and appropriateness of this method remains unknown. Future research should examine the suitability of these methods with shorter epochs, or aim to develop methods specific for separate epoch lengths.

Finally, the current study lacked a criterion measure, meaning that specific conclusions regarding which epoch length provides the most accurate representation of PA could not be made. Although direct observation may have allowed us to determine which epoch length/s were most accurate during the PE context, these findings may not have generalised to other physical activity contexts, such as non-organised or leisure time PA, as indicated by our results. It is also very difficult to differentiate between MPA and VPA using direct observation. This is because there is large inter-individual variability among adolescents in energy expenditure and activity intensity for standardized tasks such as slow walking (2.5–5.8 METs), fast walking (2.7–7.7 METs) and running (4.5–15.5 METs) [22]. Differentiating between MPA and VPA was an important component of our analyses that was not examined in previous studies [9]. Unfortunately, direct observation was not within the scope of this study, especially as we aimed to examine leisure time PA for which direct observation is impractical [37]. Future research into epoch effects could focus on testing epoch effects with such a criterion measure to determine which epoch length most accurately estimates PA. Previous work has been conducted using such procedures, but was limited in that the shortest epoch length tested was 5 s and the research was conducted only in a PE context [9]. It is unclear; however, if the application of shorter epoch lengths and shorter direct observation periods would result in more accurate PA estimates.

## Conclusion

To the authors' knowledge, this is the first study to compare epoch effects in two PA contexts with the same participants. This study has shown that adolescent boy's bouts of PA are often short, supporting the use of short epoch lengths to record all intensities of PA. Additionally, this study has shown that VPA is the most likely intensity to vary based on the choice of epoch length. Researchers interested in VPA-related research questions, such as the dose-response relationship of VPA to health outcomes or interventions designed to increase VPA, should be mindful of their choice of epoch length. If an epoch length is chosen that is too long, estimates of adolescent boys' VPA may be substantially underestimated, potentially leading to incorrect associations with health outcomes or conclusions regarding the effectiveness of interventions. Therefore, the use of a shorter epoch length, such as 1–2 s, may be most appropriate.
